# New C-Terminal Conserved Regions of Tafazzin, a Catalyst of Cardiolipin Remodeling

**DOI:** 10.1155/2019/2901057

**Published:** 2019-10-24

**Authors:** Gregory A. Shilovsky, Oleg A. Zverkov, Alexandr V. Seliverstov, Vasily V. Ashapkin, Tatyana S. Putyatina, Lev I. Rubanov, Vassily A. Lyubetsky

**Affiliations:** ^1^Institute for Information Transmission Problems of the Russian Academy of Sciences (Kharkevich Institute), 127051, Russia; ^2^Belozersky Institute of Physico-Chemical Biology, Lomonosov Moscow State University, 119991, Russia; ^3^Faculty of Biology, Lomonosov Moscow State University, 119234, Russia

## Abstract

Cardiolipin interacts with many proteins of the mitochondrial inner membrane and, together with cytochrome C and creatine kinase, activates them. It can be considered as an integrating factor for components of the mitochondrial respiratory chain, which provides for an efficient transfer of electrons and protons. The major, if not the only, factor of cardiolipin maturation is tafazzin. Variations of isoform proportions of this enzyme can cause severe diseases such as Barth syndrome. Using bioinformatic methods, we have found conserved C-terminal regions in many tafazzin isoforms and identified new mammalian species that acquired exon 5 as well as rare occasions of intron retention between exons 8 and 9. The regions in the C-terminal part arise from frameshifts relative to the full-length *TAZ* transcript after skipping exon 9 or retention of the intron between exons 10 and 11. These modifications demonstrate specific distribution among the orders of mammals. The dependence of the species maximum lifespan, body weight, and mitochondrial metabolic rate on the modifications has been demonstrated. Arguably, unconventional tafazzin isoforms provide for the optimal balance between the increased biochemical activity of mitochondria (resulting from specific environmental or nutritional conditions) and lifespan maintenance; and the functional role of such isoforms is linked to the modification of the primary and secondary structures at their C-termini.

## 1. Introduction

Cardiolipin (CL) is a phospholipid of mitochondrial membranes that directly interacts with several mitochondrial proteins to increase the efficiency of the respiratory chain and ADP/ATP exchange ([1] and references therein). It is also involved in protein import into mitochondria and modulates the mitochondrial retention and activity of many proteins and enzymes. CL oxidation triggers cell death and occurs in many pathologies [2]. Abnormal CL metabolism alters the structure of mitochondria including cristae loss and decreases the mitochondrial rate of divisions, fusions, and mitophagy. Altered CL metabolism can cause various forms of heart failure. These disturbances are particularly pronounced in Barth syndrome (BS), a rare X-linked genetic disorder with cardiomyopathy, skeletal myopathy, and growth delay [3]. The mutation associated with BS was mapped to the *TAZ* gene on the X chromosome [4]. More than 100 mutations in TAZ that induce BS have been reported [3]. They are scattered among all 11 exons of the gene and most of them are missense mutations or short indels, but frameshift and splicing site mutations, as well as large deletions of exons or the whole gene, also occur. No clear correlation between the mutation type and BS symptoms has been revealed.

Studies on human and animal models demonstrated that *TAZ* mutations decrease the level of mature CL and increase that of monolyso-CL (MLCL). The MLCL/CL ratio in the blood, together with *TAZ* mutations, is the main diagnostic features of BS. The presence of the conserved motif HXXXXD (histidine and aspartic acid separated by any four amino acids) typical of glycerolipid acyltransferases pointed to the possible involvement of tafazzin in the remodeling of nascent CL. Indeed, BS patients demonstrated low linoleic acid (C18:2) incorporation into CL in contrast to other fatty acids. Moreover, the mitochondria from rat hepatocytes and human lymphoblasts realized *ex vivo* transfer of [^14^C]linoleoyl-phosphatidylcholine to tetraoleoyl-CL, thus replacing all four acyl groups with linoleic ones [5]. Purified drosophila tafazzin efficiently transferred *in vitro* linoleic acid chains from 1-palmitoyl-2-[^14^C]linoleoyl-phosphatidylcholine to MLCL to form CL and lysophosphatidylcholine (lysoPC, LPC).

The structure of CL is unique among phospholipids; it comprises four acyl chains derived from two molecules of phosphatidic acid linked by the central glycerol backbone [6]. In most tissues, CL has one or two dominant acyl groups, which makes it structurally uniform and molecularly symmetric [7]. Residues of unsaturated fatty acids are common dominant groups. These structural properties of mature CL forms stem from its postsynthetic modification. This remodeling starts from MLCL formation through the removal of one of four acyl groups, which is catalyzed by calcium-independent phospholipase A2 in mammals. MLCL reacetylation is mediated by three enzymes: monolysocardiolipin acyltransferase, acyl-CoA:lysocardiolipin acyltransferase, and tafazzin. The substrate specificity and specific CL remodeling remain underexplored for the first two enzymes. Tafazzin is indispensable for the maintenance of the normal composition and concentration of CL. BS induction by *TAZ* mutations indicates the importance of CL remodeling and the significance of tafazzin in mitochondrial function. Tafazzins were found in all studied eukaryotes as components of the mitochondrial intermembrane compartment [7]. The topological organization of the tafazzin molecule in mitochondria remains obscure; however, it was shown to associate with 10^5^-10^6^ Da multiprotein complexes. It remains unclear which proteins comprise these complexes and directly interact with tafazzin; however, this association of tafazzin with other proteins is significant for its function.

Sequence comparison of the *TAZ* gene and several of its transcripts has revealed two alternative transcription initiation sites and several splicing variants, which give rise to several tafazzin isoforms [4]. Tafazzin sequence has no explicit similarity to other known proteins; it contains two functionally important regions: a very hydrophobic sequence of 30 amino acids in the N-terminal region, apparently, a membrane anchor, and a hydrophilic domain in the central part, apparently, interacting with other proteins. The shortest tafazzin forms lack the hydrophobic region and are likely cytoplasmic proteins, while several longer variants of alternative splicing of exons 5-7 differ by the hydrophilic domain length. It is not improbable that the diversity of the hydrophilic domains modulates their affinity to different proteins. Most common isoforms are the full-length one and the one lacking exon 5. In *Drosophila*, which also has several tafazzin isoforms, they have different intracellular localization: the dominant tafazzin-A resides in mitochondria while tafazzin-B is localized in different compartments including the mitochondria, endoplasmic reticulum, and Golgi complex.

The purified recombinant tafazzin demonstrates transacylase activity towards CL and a variety of phospholipids, such as phosphatidic acid, phosphatidylcholine, phosphatidylethanolamine, phosphatidylglycerol, and phosphatidylserine, as well as their lyso-L-derivatives. The recombinant tafazzin can transfer acyl groups of 7-19 carbon atoms with 0 to 3 unsaturated double bonds. Tafazzin function is not only the conversion of a pair of phospholipids into another pair (PL1+LPL2 → LPL1+PL2) but also keeping a balance between the PL and LPL molecules. At first sight, this wide specificity of tafazzin should level the acyl composition of all phospholipids in the corresponding membrane compartments. Actually, the *in vivo* effect of tafazzin is quite specific, primarily, towards CL of the mitochondrial compartment. Apparently, the specificity of *in vivo* transacylation is largely determined by the organization of mitochondrial membranes and the availability of acyl groups rather than by the tafazzin properties. It was suggested that the major tafazzin function is to optimize the packing of phospholipids in the membranes through the thermodynamic remodeling that facilitates dynamic conformational transitions in mitochondrial membranes [7].

A deficiency of tafazzin decreases the CL concentration, alters its acyl composition, and increases the MLCL concentration. At the same time, multiprotein complexes in the inner mitochondrial membrane degrade, which can result directly from decreased CL level or indirectly from altered conformational dynamics of the membranes. Overall, this gives an insight into the origins of abnormal functional activity of mitochondria, such as decreased membrane potential, partial oxidative uncoupling, and increased oxidative stress. A further link to the phenotypic manifestations of *TAZ* mutations is not as clear. The described molecular mechanism is universal; however, the phenotypic abnormalities in BS apply to certain tissues only. For instance, morphological abnormalities in mitochondria are observed in embryonic stem cells only after their differentiation into cardiomyocytes [8]. Apparently, highly active mitochondria with high cristae density might be most sensitive to tafazzin defects. Whatever the truth, the tafazzin deficiency does not necessarily lead to defects in the mitochondrial structural organization; rather, only the proportion of defective mitochondria increases. This effect can underlie the variation of phenotypic defects in BS.

Thus, CL is a unique dimeric phospholipid specific for mitochondria, which makes it a reliable mitochondrial marker. Tissues with high oxidative capacity such as slow-twitch skeletal and cardiac muscle have high CL content ranging from 10 to 20% of the total mitochondrial phospholipids; and this is known to be critical for mitochondrial respiration and energy metabolism [9, 10]. Specifically, CL physically interacts with and activates a large number of mitochondrial proteins including most, if not all, of the inner mitochondrial membrane enzymes, along with cytochrome c and creatine kinase [11, 12]. Actually, CL can be considered as a factor integrating components of the mitochondrial respiratory chain, which provides efficient transfer of electrons and protons [11, 12]. Not only the presence of CL but also its acylation are critical for the functional activity of mitochondria. The nature of CL acyl chains can vary between tissues; however, the dominant form in the skeletal and cardiac muscles has linoleic acid (18:2n-6). A decreased proportion of this modification can interfere with the oxidase activity of cytochrome C. The main, if not the only, factor controlling the 18:2n-6 composition of CL is tafazzin, which catalyzes the transfer of 18:2n-6 from donor phospholipids such as phosphatidylcholine, thus completing CL maturation. Accordingly, mutations in the human tafazzin gene (*TAZ*) induce BS, a form of congenital myopathy featuring structural and functional abnormalities of mitochondria, cardiac and skeletal myopathy, physical load intolerance, and increased production of reactive oxygen species [13].

The tafazzin gene was first described in 1996 as a target for mutations causing BS [4, 14]. Alternative splicing of the *TAZ* primary transcript gives rise to four different experimentally observed mRNAs: full-length (FL), lacking exon 5 (*Δ*5), exon 7 (*Δ*7), or both (*Δ*5*Δ*7) [15]. The CL acyl chain varies between cell types and tissues in the same species. The FL and *Δ*5 tafazzin forms demonstrate transacylase activity but have different topology (immersion into the membrane) [16]. It remains unclear if CL remodeling is the only function of tafazzin. *TAZ* mutations decrease the synthesis of tetra-linoleoyl cardiolipin in favor of CL molecules with a different acyl composition. This change affects the structural and functional activity of mitochondria. Analysis of the patterns and proportions of tafazzin forms in the blood of BS patients and normal subjects has demonstrated, in addition to the two functional isoforms (FL and *Δ*5), a variety of mRNA species encoding nonfunctional protein forms.

A recent phylogenetic analysis of mitochondrial proteins in mammals and birds with significantly different maximum lifespan (MLS) [17] has demonstrated that it substantially depends on the taxon-specific numeric parameter *α*, which is a component of the equation of mitochondrial metabolic rate; mtMR = *А* · *M*^(*B* − 1)/*α*^ (the dimension constant *A* will be omitted farther on). The parameter *α* characterizes the stability of proteins of the mitochondrial inner membrane. Another convenient index of mitochondrial metabolic rate is the basal rate of oxygen consumption per body weight per time (mtBRO_2_). Disregarding the constant *A*, these parameters are related by the equation mtMR^*α*^ = mtBRO_2_, where 1 ≤ *α* ≤ 8. Overall, mtMR describes the energy requirements corresponding to species living in a particular ecological niche, while *α* is determined by specific amino acid composition of mitochondrial proteins and interactions between mitochondrial membrane proteins. CL, a critical integrative component of the inner membrane, should, to a large extent, determine the value of *α* and, hence, of species-specific MLS and mtMR. As mentioned above, acyl modification of CL by tafazzin is among the main mechanisms that control the functional activity of CL. Thus, the structural and functional properties of tafazzin can be a factor of species-specific MLS and respiratory functions of mitochondria.

**Figure 1:**
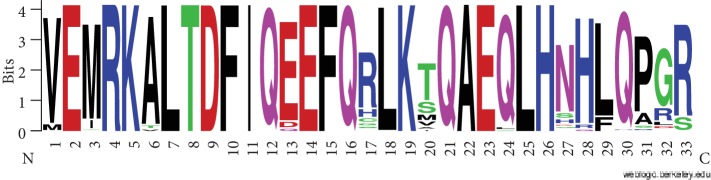
The C-terminal motif of classic tafazzins (aligned to exon 11 of human FL tafazzin).

Hereafter, tafazzin exons are numbered according to the FL isoform of human tafazzin (NP_000107; 292 amino acids). Without going into the description of classic tafazzins, note that their C-termini correspond to the motif shown in Figure 1, which was generated from the alignment of the C-terminal sequence of the mammalian proteins to exon 11 of the human FL tafazzin. The C-terminal sequences are aligned without deletions, and the secondary structure is preserved not only in mammals and other vertebrates but also in model protostomes (*Drosophila melanogaster*, *Caenorhabditis elegans*), fungi (*Saccharomyces cerevisiae*), etc. (see Figure 1 in [13]).

We bioinformatically analyzed the modifications in mammalian tafazzins in exons 5, 8–9, and 9–11. Specifically, the regions conserved among mammals have been identified in the C-terminal region of many isoforms of unconventional tafazzin as well as new species that acquired exon 5 in the tafazzin gene (apart from human and great apes; Hominidae). The first case tafazzins are referred to as unconventional (UTs), which are divided into two types (T1 and T2) distinguished by their motifs. The former motif results from the omission of exon 9 and a frameshift, while the second one results from intron retention between exons 10 and 11 and also a frameshift. The latter case when exon 5 is acquired is referred to as E5 tafazzins. In the rare cases, intron retention is observed between exons 8 and 9. These modifications have specific distribution among mammalian orders and correlate to the maximum lifespan and body weight as well as to the rate of mitochondrial metabolism. We propose the functional role of such changes.

## 2. Materials and Methods

Amino acid sequences were extracted from the RefSeq database [18] and supplemented with those from Ensembl v96 [19]. T1 isoforms of tafazzin were identified by PSI-BLAST [20] in RefSeq. The sequence ENHRADWEALQCPACARAAPGREQVSCGDSQSPD, a region of tafazzin matching in the Pacific white-sided dolphin (*Lagenorhynchus obliquidens*, isoform Х5) and the narrow-ridged finless porpoise (*Neophocaena asiaeorientalis*, isoform Х2), was used as the query. At the second iteration, the T1 set was supplemented only by tafazzins of the naked mole-rat (NMR, *Heterocephalus glaber*) as well as additional tafazzin isoforms of the giant panda (*Ailuropoda melanoleuca*) and African bush elephant (*Loxodonta africana*); these tafazzins have a lower similarity to the query. Subsequent iterations yielded no new proteins. Eventually, 50 tafazzin sequences have been identified (listed at sheet T1 of [Supplementary-material supplementary-material-1]). The *E* value cutoff was 0.005; however, the same results were obtained for the values from 0.0001 to 0.1. Hereafter, the default values were used for all unmentioned BLAST parameters.

T2 isoforms of tafazzin were identified in a similar way but using BLAST (PSI-BLAST yielded the same results). The query was the sequence PGRSSLRAAGQPQSFPSGGDSQSPD, a tafazzin region matching in the Pacific white-sided dolphin (*Lagenorhynchus obliquidens*, isoforms Х1–X4) and the narrow-ridged finless porpoise (*Neophocaena asiaeorientalis*, isoform Х1). The same results were obtained for *E* value cutoffs from 5 · 10^−5^ to 10, namely, 28 tafazzins listed at sheet T2 of [Supplementary-material supplementary-material-1] together with the query, which matches all identified regions in cetaceans. The tafazzin XP_028342714 (isoform Х1) of the sperm whale (*Physeter catodon*) corresponds to both T1 and T2 types. Its tafazzin XP_028342715 (Х2) matches the T1 and T2 types with *E* values of 10^−12^ and 2 · 10^−5^ and was assigned to T1. [Supplementary-material supplementary-material-1] includes a single tafazzin (XP_028342714) assigned to both T1 and T2 types.

E5 isoforms of tafazzin were identified by BLAST in RefSeq. The query was the human tafazzin region encoded by exons 4, 5, and 6 (underlined are the exon boundaries): TPAAADICFTKELHSHFFSLGKCVPVCRGAEFFQAENEGKGVLDTGRHMPGAGKRREKGDGVYQKGMDFILEKLNHGDWVHIFPE. The results remain unaltered for *E* value cutoffs from 10^−35^ to 10^−31^. The identified proteins contain a region between exons 4 and 6 homologous to tafazzin exon 5 of apes (Hominidae, E5 tafazzins). Higher *E* values yield amino acid regions lacking the exon 5 region that cannot be assigned to E5 tafazzins. According to [Supplementary-material supplementary-material-1], E5 and T1 types do not overlap. Some species (sooty mangabey *Cercocebus atys*, green monkey *Chlorocebus sabaeus*, crab-eating macaque *Macaca fascicularis*, black snub-nosed monkey *Rhinopithecus bieti*, and African bush elephant *Loxodonta africana*) have both types, but none of their proteins belongs to both types. Thus, these three types have a single overlap (XP_028342714) for types T1 and T2, although there is no obvious reason why the loss of exon 9 cannot be combined with the acquisition of exon 5. All identified E5 tafazzins are classic (they conform to the logo in Figure 1).

**Figure 2:**
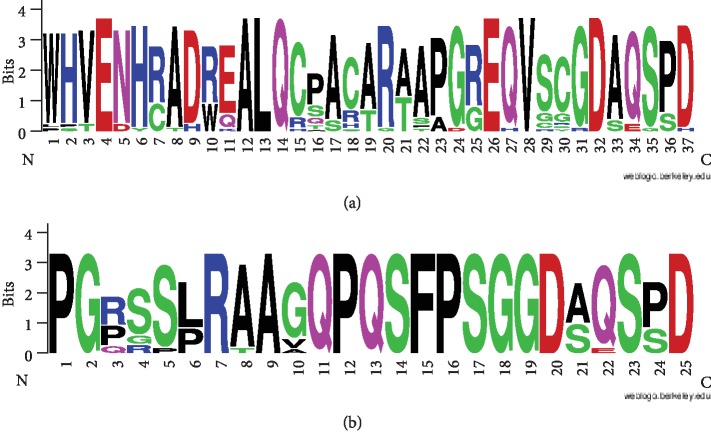
Motifs specific for unconventional tafazzin isoforms: (a) type 1 (T1) and (b) type 2 (T2).

For the generation of the sequence logos presented in Figure 2, a single region of each type (marked by an asterisk in [Supplementary-material supplementary-material-1]) was used for each species to provide for even representation of species with a different number of tafazzin isoforms. The region most similar to the above queries for T1 or T2, respectively, was selected. Sequence logos were generated using the WebLogo service [21]. The protein secondary structures were predicted by JPred4 [22].

## 3. Results and Discussion

By analyzing data available in major databases, we have identified conserved C-terminal regions missing in the classic tafazzin in the amino acid sequences of mammalian tafazzin isoforms. Their motifs (sequence logos) are shown in Figures 2(a) and 2(b), and the corresponding protein sequences are presented in [Supplementary-material supplementary-material-1]. This table also presents the taxonomy and Latin name of species to facilitate using their common names. Unconventional tafazzins (UTs) apply to tafazzin isoforms containing the first or the second motifs (T1 and T2).

The T1 motif was found in 23 mammalian species of 7 orders (or higher taxa): Afrotheria, Glires, Primates, Scandentia, Carnivora, Cetacea, and Artiodactyla, specifically in naked mole-rat (*Heterocephalus glaber*), Arctic ground squirrel (*Urocitellus parryii*), sooty mangabey (*Cercocebus atys*), green monkey (*Chlorocebus sabaeus*), crab-eating macaque (*Macaca fascicularis*), black snub-nosed monkey (*Rhinopithecus bieti*), golden snub-nosed monkey (*Rhinopithecus roxellana*), Chinese tree shrew (*Tupaia belangeri chinensis*), northern fur seal (*Callorhinus ursinus*), Steller sea lion (*Eumetopias jubatus*), California sea lion (*Zalophus californianus*), Hawaiian monk seal (*Neomonachus schauinslandi*), giant panda (*Ailuropoda melanoleuca*), Pacific white-sided dolphin (*Lagenorhynchus obliquidens*), narrow-ridged finless porpoise (*Neophocaena asiaeorientalis*), sperm whale (*Physeter catodon*), zebu (*Bos indicus*), cattle (*Bos taurus*), the hybrid of the two latter (*Bos indicus* × *Bos taurus*, NCBI:txid30522), white-tailed deer (*Odocoileus virginianus*), wild boar (*Sus scrofa*), Bactrian camel (*Camelus bactrianus*), and African bush elephant (*Loxodonta africana*).

The T2 motif coincides completely in 12 species, specifically, in nearly all cetaceans and aquatic carnivores. It was found in walrus (*Odobenus rosmarus*), Weddell seal (*Leptonychotes weddellii*), Hawaiian monk seal (*Neomonachus schauinslandi*), brown and polar bears (*Ursus arctos* and *Ursus maritimus*) as well as American black bear (*Ursus americanus*, data from Ensembl), Pacific white-sided dolphin (*Lagenorhynchus obliquidens*), killer whale (*Orcinus orca*), common bottlenose dolphin (*Tursiops truncatus*), beluga whale (*Delphinapterus leucas*), narrow-ridged finless porpoise (*Neophocaena asiaeorientalis*), and sperm whale (*Physeter catodon*). It was also found in two chiropterans, black flying fox (*Pteropus alecto*) and Egyptian fruit bat (*Rousettus aegyptiacus*), as well as in a perissodactyl, white rhinoceros (*Ceratotherium simum*).

According to MobiDB [23], the conserved UT regions overlap with long disordered regions usually up to the C-terminus. These disordered regions sometimes occur in other regions of proteins but are not typical for tafazzins without the specified T1 and T2 motifs. The identified regions cannot be aligned to domains involved in enzyme activity, which gives no grounds to assume their participation in protein attachment to the mitochondrial inner membrane or being a part of the catalytic center. Similarly, disordered regions have been found at the N termini of microtubule-binding and tubulin-sequestering proteins [24].

Although RefSeq is extensive, other databases still contain proteomes missing in RefSeq. For instance, Ensembl contains the proteome of the American black bear (*Ursus americanus*) with the tafazzin containing a region matching T1; accordingly, [Supplementary-material supplementary-material-1] was supplemented with this species and the region.

In addition to the tafazzins presented in [Supplementary-material supplementary-material-1], we have found tafazzins with the C-terminal region shared in the motifs, specifically, seven C-terminal amino acids as shown in the logo (Figure 2). These can be exemplified by the North American beaver (*Castor canadensis*), whose isoforms Х1, X2, and X5–X8 contain a region that partially matches both T1 and T2: VSFLPDSPKLSSVLPVPSDSQGTLAKVHEGCRPAPSLSAGGDAQSSD. The beaver can also be assigned to E5. Similarly, all isoforms (X1–X4) of the European rabbit (*Oryctolagus cuniculus*) include shortened T1 or T2; these are similar regions LPQGCGPTVSLSSGGDAQSPH (isoforms Х1 and X4), GCGPTVSLSSGGDAQSPH (Х2), and LPQGCGLSSGGDAQSPH (Х3), which allow us to call such proteins *shortened* UTs. Isoform X1 in naked mole-rat (*Heterocephalus glaber*) also contains the sequence GDAQGPD. Also, its T1 is represented by isoforms X2 and X3 while classic tafazzins include isoforms X4 and X5. The list of such examples goes on.

There are regions with very weak similarity to T1 or T2. For instance, isoform X1 of the Colombian white-faced capuchin (*Cebus capucinus*) is similar to T1: ALWRPDAGGAEREAAARDRVEHRDFLAPRH; isoform X2 of the domestic sheep (*Ovis aries*) is similar to T2: VSFSLGLSSPFSLGLSSP; tafazzin of the dromedary (*Camelus dromedarius*) is similar to T2: VSSSPRQSCSCPSPSSP. To our knowledge, the capuchin has no classic tafazzin.

It is of interest that the regions GDAQSPD, GDAQSSD, GDAQSPH, GDSQSPD, GDAESPD, and GDAQGPD corresponding to the last seven positions in Figure 2 occur exclusively in unconventional tafazzins including shortened ones. Among 4 million proteins in RefSeq, there are only three exceptions, XP_007521742, XP_004712810, and XP_016049842 of European hedgehog (*Erinaceus europaeus*) and lesser hedgehog tenrec (*Echinops telfairi*), that are not tafazzins but include regions 2, 4, and 5 above. Specifically, the exact GDAQSPD sequence is present in as low as 28 mammalian proteins: 23 T1s and 5 T2s. GDSQSPD is present in 17 mammalian proteins: 4 T1s, 12 T2s, and 1 nontafazzin XP_016049842 of the hedgehog (*Erinaceus europaeus*). GDAQSSD is present in 20 mammalian proteins: 6 T1s, 8 T2s, 6 shortened UTs of the beaver (X1, X2, and X5–X8), and 1 nontafazzin XP_007521742 of the hedgehog. GDAESPD is present in 7 mammalian proteins: 4 T1s, 2 T2s, and 1 nontafazzin XP_004712810 of the tenrec (*Echinops telfairi*). GDAQSPH is present in 5 mammalian proteins: 1 T1 of Arctic ground squirrel (*Urocitellus parryii*) and 4 shortened UTs of the European rabbit (*Oryctolagus cuniculus*, X1–X4). GDAQGPD is present in 2 mammalian proteins: 2 T1s of the naked mole-rat (*Heterocephalus glaber*). RDAQSPD is present in 3 T1s of the African bush elephant (*Loxodonta africana*) and in more than a hundred nontafazzins of the family of sister chromatid cohesion protein PDS5 homolog A. Thus, these six regions largely mark unconventional tafazzins.

Hereafter, *patterns* refer to tafazzin regions specified in Materials and Methods as queries. It is natural to define the threshold segregating true T1 and T2 proteins from those with only a remote resemblance to these types. In the case of T2, at least 19 out of 25 amino acids have to match, i.e., more than 3/4. In the case of T1, it seems that true regions match at least 2/3 of the pattern (more than 22 out of 34 amino acids), although some isoforms with a lower similarity were included in the list in [Supplementary-material supplementary-material-1]. For instance, there is a region with a 50% identity (17 out of 34 amino acids) in isoform X14 (XP_023396534) of the African bush elephant (*Loxodonta africana*); however, another isoform of this species (X10) used in the logo generation has more than 2/3 of matches (23 out of 34). The challenges of such a threshold definition are shown below. The prairie deer mouse (*Peromyscus maniculatus bairdii*) represented in Ensembl but not in RefSeq has a sequence matching almost a half of the first part of the T1 pattern (16 out of 34 amino acids). Such shortened T1 is typical of many rodents (the number of unconventional isoforms in RefSeq is given in parentheses): house mouse (*Mus musculus*, 6), Gairdner's shrewmouse (*Mus pahari*, 3), Ryukyu mouse (*Mus caroli*, 2), Chinese hamster (*Cricetulus griseus*, 4), Golden hamster (*Mesocricetus auratus*, 2), lesser Egyptian jerboa (*Jaculus jaculus*, 2), and guinea pig (*Cavia porcellus*, 1). At the same time, the alignment of the region of tafazzin of the prairie deer mouse demonstrates a convincing similarity, and the absence of such tafazzins in [Supplementary-material supplementary-material-1] is due to the similarity shortness, which we consider significant. The asterisk in the prairie deer mouse sequence indicates the stop codon:

Query: ENHRADWEALQCPACARAAPGREQVSCGDSQSPD

Subject: ENHRADREALQCTPCA^∗^

Note that arginine at position 7 is not uncommon in mammals.

The absence of the major part of the patterns in shortened tafazzins does not allow us to assign them to UTs, although the threshold similarity length has not been defined explicitly.

In addition, new tafazzin isoforms that contain exon 5 have been found in the following orders (or higher taxa): Afrotheria, Glires, Primates, Carnivora, Cetacea, Artiodactyla, and Chiroptera. Previously, such E5 tafazzins have been found in hominids (*Homo sapiens*, *Pan paniscus*, *Pan troglodytes*, *Gorilla gorilla*, and *Pongo abelii*). Apart from these, we have identified E5 tafazzins in many Old World monkeys (Cercopithecidae): *Cercocebus atys*, *Chlorocebus sabaeus*, *Macaca fascicularis*, *M. mulatta*, *M. nemestrina*, *Mandrillus leucophaeus*, *Papio anubis*, *Theropithecus gelada*, and *Rhinopithecus bieti* as well as in the northern greater galago *Otolemur garnettii*. Occasional E5 tafazzins can be found beyond primates: in rodents (*Ochotona princeps* and *Castor canadensis*), laurasiatherians (*Pantholops hodgsonii*, *Phyllostomus discolor*, *Balaenoptera acutorostrata*, and *Puma concolor*), and afrotherians (*Loxodonta africana*). All identified E5 proteins are given in [Supplementary-material supplementary-material-1] (sheet E5).

Complementation test in yeast has demonstrated the functional activity only for the *Δ*5 variant, which raises the question as to why exon 5 was preserved in evolution [25]. The full-length human tafazzin proved to complement the deletion of *TAZ* gene in *Drosophila* [16]. Schlame claimed that exon 5 could be found in the *TAZ* gene only in primates [7]; however, we have found many new instances of this exon.

**Figure 3:**
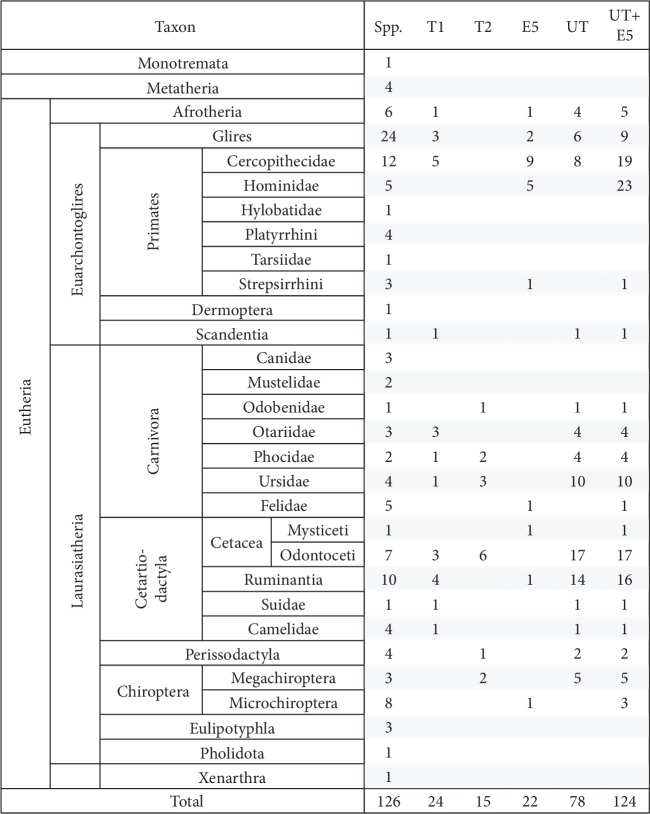
Distribution of species containing each unconventional type or E5 tafazzins and their total number per taxon. The following indices are given for each taxon (left to right): total number of considered species, numbers of species with T1, T2, and E5, the total number of unconventional tafazzins, and this number supplemented with E5 tafazzins.

Figure 3 shows the number of considered species, the number of species containing each unconventional type or E5 tafazzins, and their total number per taxon (these data are given in more detail in [Supplementary-material supplementary-material-1]).

In isolated cases, classic tafazzins conforming the description in Introduction preserve the intron between exons 8 and 9; such tafazzins will be referred to as CT+. Nine such cases have been found in RefSeq in the following primates: *Homo sapiens*, isoforms X2, X3, and X5 (XP_006724900, XP_016885250, and XP_024308199, respectively); bonobo (*Pan paniscus*), X2 (XP_008950941), Sumatran orangutan (*Pongo abelii*), X1 (XP_024096396), black-capped squirrel monkey (*Saimiri boliviensis*), X1, X2, and X5 (XP_010330095, XP_010330096, and XP_010330098), and chimpanzee (*Pan troglodytes*), X5, (XP_016798103). Specifically, the glycine (underlined) at the boundary of these exons, WHVGMND, is replaced with one of the following regions corresponding to a 117 bp intron insertion: GEPGDGDREMASGVGGLGLPLVPGCPAPPHVWPSVHCAAG (human, bonobo, and chimpanzee), GEPGDGDREMASGVGGLGVPLVPGCPAPPHVWPSVHCAAG (orangutan), and GEPGDGDRDKASGVGSLGLPLVPGCPAPPHVWPFVHCAAG (squirrel monkey).

Noteworthily, this intron retention exists in the human and orangutan but is missing, e.g., in the gorilla.

**Figure 4:**
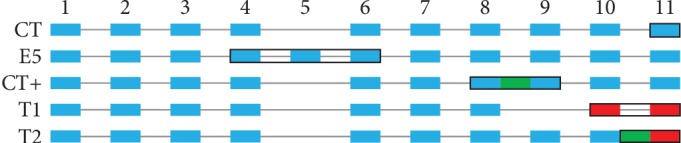
The exon-intron structures of the considered tafazzin types. Usual exons (i.e., as in CL) are depicted by blue rectangles, retained introns are shown in green, and frameshifted exons are shown in red. The black outlines emphasize the distinguishing features of the tafazzin types.

The exon-intron structures of the discussed tafazzin types are schematically shown in Figure 4.

As already noted, the current work is devoted to the bioinformatic study of tafazzin isoforms. Experimental verification that such tafazzin isoforms are actually expressed will be performed in a separate work.

**Figure 5:**
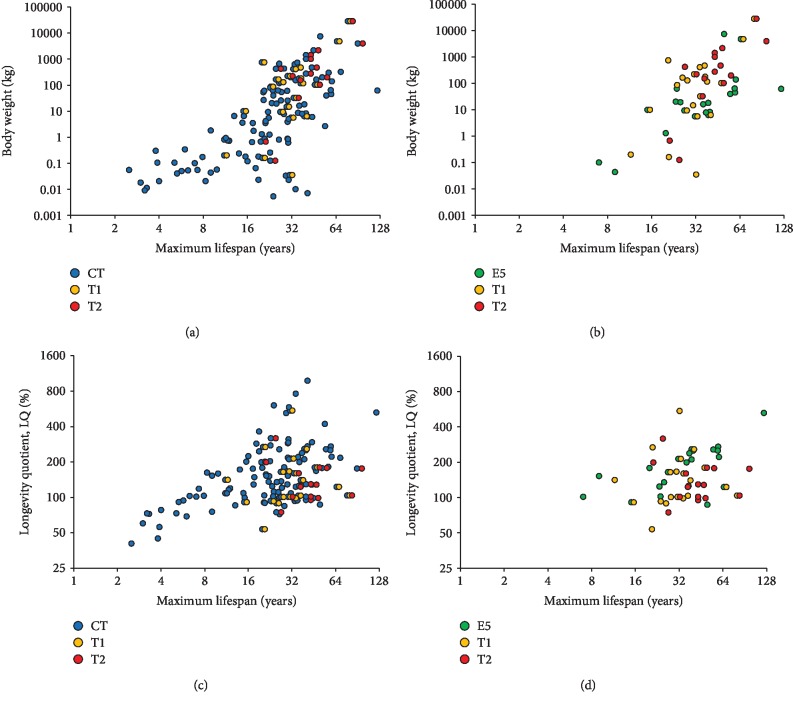
Distribution of mammalian tafazzins over lifespan and body weight (a, b) or longevity quotient (c, d) for classic tafazzin (CT) and unconventional T1 and T2 ones (a, c) or E5, T1, and T2 tafazzins (b, d).

Scatter plots for maximum lifespan (MLS, years) vs. body weight (M, kg) were generated for all classic and identified unconventional tafazzins (Figure 5). MLS data were retrieved from the AnAge database [26]. The diagram in Figure 5(a) demonstrates that species with T2 and, to a lesser extent, T1 tend towards high body weights relative to those with the classic tafazzin. Specifically, T2 is observed in the case of body weights exceeding 100 kg except large bats: Egyptian fruit bat (*Rousettus aegyptiacus*, 125 g) and black flying fox (*Pteropus alecto*, 672 g) as well as narrow-ridged finless porpoise (*Neophocaena asiaeorientalis*, 32.5 kg). T1 is observed in the case of body weights exceeding 5.5 kg with the exception of naked mole-rat (*Heterocephalus glaber*, 35 g), blind mole-rat (*Nannospalax galili*, 160 g), and tree shrew (*Tupaia chinensis*, 200 g). Е5 (Figure 5(b)) is observed in the case of body weights exceeding 5.5 kg with the exception of pale spear-nosed bat (*Phyllostomus discolor*, 43 g), American pika (*Ochotona princeps*, 100 g), and northern greater galago (*Otolemur garnettii*, 1.3 kg). Similarly, MLS vs. longevity quotient (LQ) was considered (Figures 5(c) and 5(d)). T2 is characterized by a long lifespan (20 years or more even in the wild) and average LQ, while E5 features long lifespan and high (Hominidae) or average (the rest in Figures 5(c) and 5(d)) LQ. T1 has a wide range of LQ values but tends towards average LQ and long lifespan (more than 11 years).

**Figure 6:**
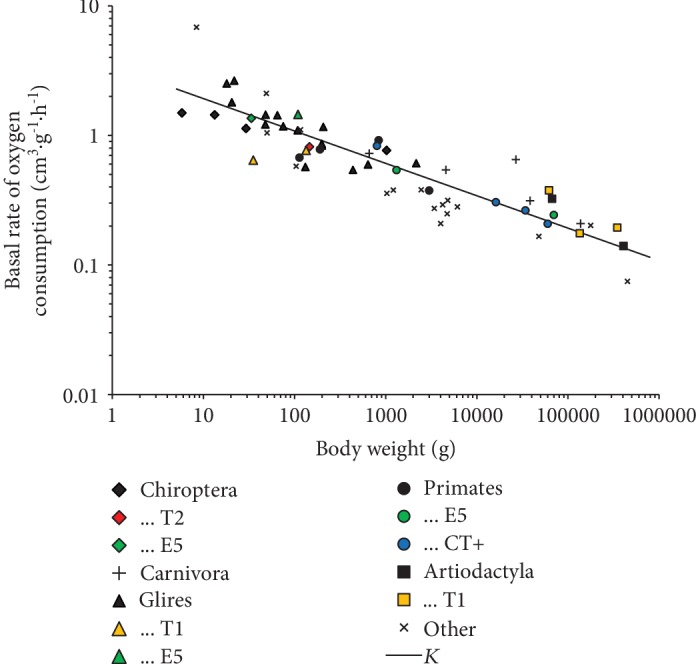
Oxygen consumption by mammals. Unconventional tafazzins T1 and T2 as well as E5 and CT+ are marked in yellow, bright-red, bright-green, and blue, respectively (the data for the CT+ species are from [28]). The line labeled *K* is the Kleiber relation.

Not much data are available on the rate of mitochondrial metabolism for the considered species. These include the data on the mitochondrial metabolic rate (mtMR) [17], basal rate of oxygen consumption (BRO_2_) [27, 28], and (the most complete) mass-specific basal metabolic rate (msBMR) from the AnAge database [26] and elsewhere [29]. Several indices are available for certain species, which allowed us to reduce the available data to a single characteristic (Figure 6). The figure suggests that unconventional tafazzin isoforms focus on the optimal balance between the increased biochemical activity of mitochondria related to environmental or nutritional conditions and longevity maintenance. These unconventional tafazzins form two clusters with a significant difference in the body weight; the first one includes three artiodactyls (cattle, wild boar, and white-tailed deer; yellow squares), chimpanzee, orangutan, and human (neighboring bright-green and blue circles; according to E5 and CT+); while the second one includes the naked and blind mole-rats (yellow triangles), the microbats (Microchiroptera; 33.5 and 146 g; bright-green and bright-red diamonds), American pika (bright-green triangle), New World monkeys (squirrel monkey, blue circle above the curve; according to CT+), and northern greater galago (bright-green circles). In the second cluster, the body weight is nearly 100 times lower; however, the rate of oxygen consumption per body weight is 4-5 times higher. This is in a good agreement with the Kleiber equation $\dot{V}\left(O_{2}\right)/m=3.42\cdot m^{-0.25}$ [30] presented as a straight line in the figure. One can propose that the emergence of UTs in addition to E5 was a response to the increased mass-specific oxygen consumption considering that it is found in aquatic mammals, large bats, and white rhinoceros.

*(1) Conservation of Cardiolipin Synthase and Variability of Tafazzin*. Cardiolipin synthase 1 encoded by the gene CRLS1 (ENSG00000088766) in human is highly conserved. A single isoform exists in most species. The reaction catalyzed by it yields a variety of cardiolipins whose transformation is mediated by the classic and unconventional isoforms of tafazzin. One can propose that these isoforms modulate the acyl composition of cardiolipins as a function of environmental conditions.

*(2) The Possible Relationship between T1 and T2*. In addition to the discussed above sperm whale tafazzin XP_028342715 (X2) to a different extent applying to the T1 and T2 types, there is another sperm whale protein XP_028342714 (X1) fully applying to T2 and satisfactorily applying to T1. It is the only known tafazzin with a complete T2 motif preceded at a distance of 16 amino acids by an almost complete T1 motif (lacking the terminal GDSQSPD). This isoform was assigned to both types, T1 and T2. These three isoforms illustrate a possible transition from the “intermediate” T1 type to the “new” T2 type. Specifically,


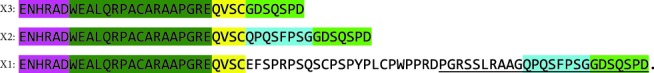


Here, X3 is a typical T1; X2 is a T1 with insertion from T2 (turquoise); and X1 is a T1 with an insertion converting it into T2 (T2-specific motif is underlined). Apparently, the loss of exon 9 is more common than the intron fixation here. Coupled with the high number of T1 tafazzins, this points to the emergence of T2 after T1.

*(3) Relationship between UT and Exons*. In the classic tafazzin, the translation of exon 10 starts in phase 0 (i.e., the first exon nucleotide is the first codon nucleotide). The T1 tafazzin results from skipping exon 9 (see Figure 4) so that the spliced out region is not a multiple of three. After exon 9 splicing, the translation of exon 10 starts in phase 1 (the first nucleotide of the exon is the second nucleotide in the codon) and the first 26 amino acids of T1 motif are synthesized. The remaining 8 amino acids of the motif result from the translation of the subsequent exon eleven (also in phase 1 since the length of exon 10 is a multiple of three).

This mechanism can be demonstrated on mouse tafazzin isoforms from Ensembl. The isoform ENSMUSP00000065270 corresponds to the classic tafazzin, while the other one (ENSMUSP00000134745) lacks exon 9 (ENSMUSE00000209157). In the first case, exon 10 translation yields amino acid sequence KITVLIGKPFSTLPVLERLRAENKSA; in the second case, ENHRADWEALQYTPCA, which corresponds to the onset of T1 motif. The mouse protein terminates here due to a stop codon; in the absence of it, the following sequence corresponds to T1 motif. This can be illustrated by the human FL isoform of tafazzin ENSP00000469981. After deletion of exon 9 (ENSE00003724812) from its transcript (ENST00000601016), the amino acid sequence corresponding to exon 10 and beginning of exon 11, KITVLIGKPFSALPVLERLRAENKSAVEMRKALT…, is replaced with ENHCADREALQCPACTRAAPGGEQVGCGDAESPD…, which corresponds to T1 motif. No exon 9 splicing has been reported for human; however, such proteins were experimentally demonstrated in mouse (e.g., Q810E8 in UniProt). It is not unlikely that the stop codon of the primary transcript is edited and translated as an amino acid in certain species.

Similarly, it can be shown that T2 results from intron retention between exons 10 and 11 (see Figure 4). This can be illustrated by two tafazzin isoforms of the polar bear. The first transcript (ENSUMAT00000031820), a classic tafazzin, has no introns; and translation of exons 10 and 11 generates the classic C-terminus: KITVLIGKPFSALPVLERLRAENKSAVEMRKALTDFIQEEFQRLKTQAEQLHNQLQRGR. In the second transcript (ENSUMAT00000031828), intron retention between exons 10 and 11 gives rise to the C-terminus with a typical T2 motif: KITVLIGKPFSALPVLERLRAENKSAVSCLSPLYHPPFPGLPCSCLSLSRHLQPPRAPGSSS**PGPGSPRAAVQPQSFPSG****GDAQSSD**…. The sequence encoded by the retained intron is underlined and T2 motif is in bold. Notice that, similar to T1, exon 11 is translated in phase 1 rather than the natural phase 0, which explains the coincidence of the last seven amino acids in these two motifs (see Figure 2).

**Figure 7:**
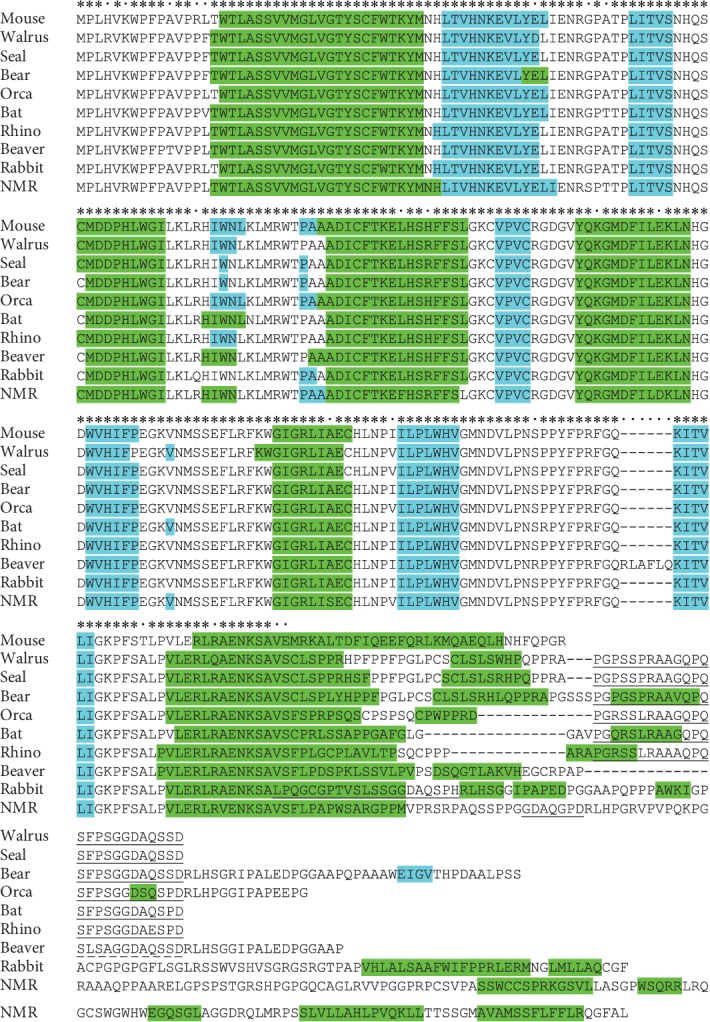
Secondary structure of tafazzin. Helices and extended regions predicted by JPred4 are marked green and blue, respectively. Mouse, CT; walrus, seal, bear, orca, bat, and rhino, T2; beaver, rabbit, and NMR, shortened UT where underlined regions support their assignment to UT (beaver and NMR also have different isoforms listed in [Supplementary-material supplementary-material-1]). Abbreviations: mouse: NP_852657.1, isoform 2 (*Mus musculus*); walrus: XP_004414556.1, X1 (*Odobenus rosmarus*); seal: XP_006739411.1, X1 (*Leptonychotes weddellii*); orca: XP_012394911.1, X1 (*Orcinus orca*); bear: XP_026344949.1, X1 (*Ursus arctos*); bat: XP_015979168.1, X1 (*Rousettus aegyptiacus*); rhino: XP_014653014.1, X1 (*Ceratotherium simum*); beaver: XP_020040765.1, X1 (*Castor canadensis*); rabbit: XP_017194047.1, X1 (*Oryctolagus cuniculus*); NMR: XP_004875054.1, X1 (*Heterocephalus glaber*). Entirely conserved positions are labeled with asterisks.

*(4) Special Features of C-Termini of UTs*. The mechanism of UT realization, i.e., the functional role of the revealed conserved C-terminal regions of tafazzin, is of great interest. In this context, it should be noted that the C-terminal secondary structure differs in UTs and classic tafazzins (CTs) (Figure 7). For instance, the house mouse CT (ENSMUSP00000065270.6) has a single long helix at the C-terminus, while in shortened UTs it is broken into two (walrus, beaver, and rabbit) or more (naked mole-rat) parts. One can propose that these C-terminal helices in UTs do not interact with the membrane since they are rich in polar amino acids. Specifically, the C-terminus of these tafazzins following the RAENKSA motif contains 2-5 times more polar amino acids, which decreases the C-terminal hydrophobicity.

*(5) The Specificity of UT Taxonomic Distribution*. UTs demonstrate highly uneven distribution in Euarchontoglires and Laurasiatheria. This is systemically shown in Figure 3 and briefly exemplified here. UTs are not found in monotremes and marsupials and are rare in afrotherians (1 out of 6 = 17%); this is also true for Е5. A similar UT distribution is observed in Euarchontoglires (9/51 = 18%), specifically, in Glires, Old World monkeys, and tree shrew; Е5 is more common (17/51 = 33%) in the same orders and families plus hominids and lemuriform primates (Strepsirrhini). UTs are found in Laurasiatheria in a much higher proportion, specifically, in pinnipeds, bears, toothed whales, ruminants, swines, camelids, perissodactyls, and fruit bats, while the proportion of E5 is much lower (4/62 = 6%). Neither UT nor E5 has been found in insectivores, pangolins, and anteaters and sloths. Usually, one of the types (T1 or T2) is represented in a family excluding earless seals and bears (Carnivora) and toothed whales. T2 was found in the polar, American black, and brown bears, while their remote relative, the giant panda, has Т1. Т2 was also found in fruit bats but is missing in considered microbat species of the *Myotis* genus.

*(6) UTs and MLS*. The presence of UTs somewhat correlates with MLS as indicated by the examples below. Among long-lived rodents, unconventional T1 is found in the naked and blind mole-rats but is missing in the Damaraland mole-rat. No UTs have been found among other rodents except for the Arctic ground squirrel. Among primates, T1 was found only in certain Old World monkeys with low MLS as well as in a close relative of primates, the tree shrew (Euarchonta). Among afrotherians, T1 was found only in the African bush elephant. Among artiodactyls, T1 was found in species with both high (zebu, cattle, and Bactrian camel) and lesser MLS (white-tailed deer and wild boar). Many aquatic mammals with high MLS proved to have T1 or T2 or both. Overall, many long-lived species belong to orders where unconventional or E5 tafazzins were identified (primates, carnivores, perissodactyls, and cetartiodactyls).

*(7) UTs and Body Weight*. UTs demonstrate an interesting distribution across taxonomic groups as a function of body weight. Irrespective of taxonomic groups, considered mammals with body weight exceeding 1000 kg had T1 (sperm whale and elephant) or T2 (walrus, killer whale, beluga whale, and sperm whale) (Figure 3) with a single exception: no UT has been found in the common minke whale; however, the group of baleen whales remains underexplored and its only classic tafazzin is marked as a low-quality protein in NCBI.

In the range from 500 to 1000 kg, T2 has not been found among considered species. In ruminants, T1 was found only in the cattle and zebu (livestock), which can be attributed to increased biodiversity after natural selection was replaced with artificial one, whose rate is much higher [31]. Only classic tafazzin was found in the wild yak, bison, and wild water buffalo.

Nearly a half (7/15 = 47%) of species with T2 fall into the range from 100 to 500 kg; these include cetaceans and carnivores. In tylopods, T1 was found in the domestic Bactrian camel but is missing in the wild Bactrian camel, which are considered different species [32]. This agrees with the above pattern for domestic and wild ruminants. In perissodactyls, UTs are absent in the common donkey, domesticated horse, and Przewalski's horse. Their tafazzins have RAENKSA sequence at the end of exon 10; however, the following sequences does not allow them to be assigned to T2.

In monkeys weighing less than 100 kg, T1 is found in about a half of the Old World monkeys (5/12 = 42%) with the terminal sequence GDAQSPD (except isoform X5 in the sooty mangabey *Cercocebus atys*); apparently, it competes with the classic monkey tafazzin with the exon 5 insertion. All hominids have only the classic tafazzin (with the exon 5 insertion).

UTs are found in marine carnivores with the body weight from 165 to 1012 kg, i.e., within 2- to 3-fold variation from 500 kg; the latter value corresponds to the optimal balance between heat exchange and food resources [33]. No UTs were found in mustelids and baleen whales, whose body weight differs from 500 kg by order of magnitude, which can reflect different energy expenditures related to the food resources or a different evolutionary pathway. The manatee (Afrotheria) weighing 322 kg is the exception.

*(8) UTs and Evolution of Species*. The relationship between UTs and evolution of species requires further analysis. However, the following observations deserve to be mentioned. No UTs were found in bats except T2 in two species that lack echolocation. Microbats followed their own evolutionary pathway resulting in decreased body size, special skills (echolocation, etc.), and improved flight performance [34]. Also, they have a higher metabolic activity owing to genes of the oxidative phosphorylation pathway and DNA repair efficiency [35]. In Old World primates, UTs are absent in hominids, T1 is found in monkeys, and both taxa have E5. UT and E5 are missing in New World monkeys. UTs are absent in lemuriformes. Apart from T1, which occurs in many mammals, more than half of marine mammals have T2. UTs have not been found in monotremes and marsupials as well as in early diverged placentals (Hoffmann's two-toed sloth and armadillo); T1 UT was found only in the African bush elephant among afrotherians. Thus, one can conclude that UTs emerged late in evolution: they are absent in monotremes (218 MYA), marsupials (169 MYA), anteaters and sloths (99 MYA), and afrotherians (94 MYA) excluding the African bush elephant and later in insectivores (81 MYA) and pangolins (74 MYA) [36].

## 4. Conclusions

A wide but specific distribution of tafazzin (a cardiolipin remodeler) with altered C-terminus or intron insertions across orders and other taxa was demonstrated in Euarchontoglires and Laurasiatheria. Specifically, we have found conserved regions closer to the C-terminus in many unconventional isoforms, rare cases of intron retention between exons 8 and 9, and new species that acquired exon 5 in the tafazzin gene (apart from Hominidae). The C-terminal regions result from a frameshift relative to the full-length *TAZ* transcript after skipping exon 9 or retention of the intron between exons 10 and 11. The altered ratio between tafazzin isoforms can cause severe diseases such as Barth syndrome. These alterations demonstrate specific distribution among mammalian orders. The dependence of the species maximum lifespan, body weight, and mitochondrial metabolic rate on the alterations has been demonstrated. Arguably, unconventional tafazzin isoforms provide for the optimal balance between the increased biochemical activity of mitochondria (resulting from specific environmental or nutritional conditions) and lifespan maintenance, and the functional role of such isoforms is linked to the modification of the primary and secondary structures of their C-termini.

## Data Availability

All data used to support the findings of this study are included in the article and the supplementary file.

## Supplementary Materials

Supplementary Materials Table S1: the sheet “species” covers the distribution of identified unconventional isoforms of tafazzin among 125 mammalian species that have an ortholog of the human tafazzin (GeneID 6901) according to Entrez Gene database [37]. Column 1 indicates taxon ID in NCBI taxonomy database [38]. Column 2 shows a brief species taxonomy within the order of mammals; column 3, species scientific name; columns T1, T2, and E5, numbers of the corresponding tafazzin isoforms identified. The sheets T1, T2, and E5 list the corresponding isoforms identified. In these sheets, column 1 indicates protein ID in RefSeq; columns 2 and 3 replicate those from the “species” sheet; column 4 specifies the isoform number; asterisk in column 5 indicates proteins used in the logo generation (Figure 2). The last column presents the protein regions. The central part in sheet E5 contains hominid proteins where E5 was previously identified elsewhere.
